# “Hidden” and Diverse Long-Term Impacts of Exposure to War and Violence

**DOI:** 10.3389/fpsyt.2019.00975

**Published:** 2020-01-17

**Authors:** Boris Drožđek, Jan Rodenburg, Agnes Moyene-Jansen

**Affiliations:** ^1^PsyQ Psychotrauma, Parnassia Group, Eindhoven, Netherlands; ^2^GGz Momentum, Den Bosch, Netherlands

**Keywords:** trauma, stress, posttraumatic stress disorder, war, violence, diagnosis, resilience, long term

## Abstract

Nowadays, the PTSD diagnosis is often a prerequisite for the survivor’s access to specialized treatment services and for obtaining legal recognition or financial compensation when exposed to violence. However, some survivors do not meet all necessary criteria for the PTSD diagnosis, particularly not in the long term. Therefore, they run the risk of being misdiagnosed, inadequately helped or undertreated, and may remain legally unrecognized and unprotected. In this article the “hidden” long-term impacts of exposure to war and violence, beyond the PTSD diagnosis, are presented, discussed, and illustrated with case presentations. They include dissociative states, attachment problems, personality changes, guilt, shame, rage, identity issues, moral injury, substances abuse, damaged core beliefs, and bodily sensations linked to stress activation. These phenomena are not persistent, but fluctuate over the survivor’s life trajectories. Moreover, the “hidden” impacts are framed within theoretical models for understanding long-term impacts of exposure to violence. The models help us grasp the dynamics of interactions between resilience, psychological damage, context and time. These interactions are non linear, and contingently result in development of psychopathological phenomena when reaching a threshold during a process of accumulating potentially traumatic experiences over a survivors’ lifetime. Understanding psychological impacts of exposure to violence as a spectrum of interchangeable phenomena over a lifetime, and learning to recognize the “hidden” manifestations of psychological trauma will help to improve mental and legal assistance to the survivors both on a short and long term.

## Introduction

Exposure to life-threatening events can cause psychological problems, and, at some point, most people will be exposed to one or more of such events in their lives (1). The posttraumatic stress disorder (PTSD) is a diagnostic category (2) constructed with the aim to capture these problems in individual survivors of trauma. According to a body of research (3, 4), the prevalence of PTSD varies from 1 to 40%, depending on the populations examined and the types of traumatic experiences they have been submitted to. More specifically, in refugee survivors of war and violence, prevalence of PTSD ranges from 12 to 34% (5). PTSD typically co-exists with other forms of psychopathology, and 90% of survivors with PTSD have at least 1 lifetime comorbid disorder (3). The most prevalent comorbid conditions are depression, alcohol abuse or dependence, and another anxiety disorder, and there is increasing evidence for co morbidity with a borderline personality disorder (6). In 38.2% of military and 15.3% of civilian cases, PTSD can occur in a delayed form, years after exposure to traumatic experiences (7).

Research with the US veterans population (8) shows that many veterans do not seek treatment for PTSD. They perceive social stigma for suffering from a mental health adversity as the main barrier to seek assistance, together with institutional factors, like having a perception of not “fitting into” the care system, having doubts about treatment staff’ sensitivity and skills, and experiencing logistic barriers. Further, the more severe mental health problems are, the more difficulty veterans and other trauma survivors seem to have in navigating the complexities of accessing care (9, 10). Research on accessibility of care for trauma survivors among immigrants shows that this heterogeneous group also faces substantial obstacles in accessing health care services (11). Based on a longstanding clinical experience in the Netherlands and recent research in several European countries (12), we hypothesize that the barriers for the refugee population in host countries are even higher, as many treatment facilities are reluctant to assist them because of communication problems (i.e. interpreters should be paid by the facility), and a lack of experience with assisting clients with other cultural backgrounds. Also, the highly specialized trauma treatment facilities often only accept clients presenting themselves with PTSD, and exclude those suffering from other mental health adversities when exposed to traumatic experiences. The treatment itself is often following a trauma-focus protocol and the intervention usually shows a lack of sensitivity for contextual issues in daily lives of clients.

Although the PTSD framework has helped to understand the impacts of exposure to traumatic experiences, and has produced significant advances in the knowledge about aetiology, prevalence and treatment of post traumatic psychological phenomena, it has also been criticized for promoting a medicalised and a reified perspective of post traumatic psychological impacts, and for being culture-insensitive (13, 14). The dominant emphasis on PTSD in current research and literature, has also been denounced for simplifying human reactions to traumatic experiences to a binary distinction between pathology (PTSD) versus absence of it (15), while at the same time, clinical practice shows that survivors of trauma can present with a wide range of psychopathological phenomena over their lifespans. These phenomena mirror different aspects of survivors’ (mal)adjustment to post traumatic impacts. These aspects, the “hidden” impacts of trauma, are still insufficiently understood, and should receive a more prominent place in future research.

## Framing Psychological Impacts Beyond Ptsd

Over the past decades, several theories and conceptualizations have been developed to come to grips with the complexity of psychological posttraumatic impacts beyond the PTSD paradigm. These models consider the complex interplay of multiple and reciprocal psychosocial, policy-related and ecological factors that simultaneously impact on the key domains of survivors’ lives (16), and subsequently determine their psychological reactions.

The concept of cumulative trauma (17) introduced the dimensions of time and the interactive relationship between an individual and his/her ecological surroundings into the discussion regarding trauma, thereby transforming the event (traumatic experience) into a process over the survivor’s life trajectory.

The concept of sequential traumatization (18) stressed that ongoing changes in the environmental/historical context of the individual survivor interact with traumatic experiences over time, recognizing that the quality and quantity of traumatic sequences can differ in various contexts and at different times across a lifespan.

Conservation of Resources (COR) theory (19, 20) viewed loss of resources when exposed to traumatic experiences as the key component in the process leading to development of mental health problems. According to this theory, individuals accumulate resources in order to accommodate, withstand, or overcome threats. These resources are personal, such as self-esteem, material, such as money, and conditional, such as status and social support. Stressful or traumatic events consume these resources, and augment one’s sensitivity to subsequent stressors. COR theory analyses a flux of resources at times of stress, and provides a framework for comparing the relative loss of resources with risk of adverse mental health outcomes.

As the PTSD concept is criticized for a lack of cultural sensitivity, the framework of Social Constructivism (21) was developed. It stressed the importance of understanding survivors’ worldviews or life experiences as they are rooted in their specific cultural contexts (the insider, emic perspective), instead of looking for universal truths (the outsider, ethic perspective) while trying to understand the complexity of posttraumatic damage. It also accentuated the role of the process of giving meaning in mediating human responses to life adversities. The framework pointed to the importance of focusing on local idioms of distress, identification of local mental health concerns and priorities, understanding the effects of organized violence on multiple levels of the survivor groups cosmology or world views (in relation to the family, community, and society), understanding of local patterns of help-seeking behaviour, and last but not least, identifying local resources that can promote healing and adaptation, and thereby create context relevant intervention strategies.

The Integrative Contextual Model (22, 23) for understanding, and assessment of post trauma mental health consequences merged the developmental and the ecological perspectives. This model perceives a mental health problem as a consequence of imbalance between sources of psychological resilience and damage, which are rooted in all levels of survivor’s ecological environment. This balance may change over time, as the dynamic of the relationship between mental health problems and context is bidirectional. In order to understand the complexity of this relationship, one should be guided by a string of causation principles and grasp the logic of fluctuation of psychopathology over survivor’s life trajectory.

Bonanno et al. (24) and Bonanno and Mancini (15) challenged the assumption that aversive life events produce a single homogeneous distribution of change over time, and identified a heterogeneity of responses in individual survivors. These unique trajectories of adjustment in the aftermath of potentially traumatic events (PTEs) are: resilient trajectory, gradual recovery, delayed reactions, and chronic dysfunction. These authors advocate a dimensional rather than a categorical structure of individual responses after trauma.

The Adaption and Development After Persecution and Trauma (ADAPT) model (25) helped understanding comorbid psychopathological patterns in relation to the distinct pathways arising from disruptions of a combination of the five core psychosocial pillars after trauma. These pillars are: safety, integrity of bonds and networks, systems of justice, roles and identities, and systems of meaning and coherence. It suggested that identifying the links that connect the disrupted psychosocial domains with the manifestations of psychopathology of the individual and the capacity of the individual and its collectives to mount effective adaptive responses, is key to achieving a comprehensive understanding of the survivors’ needs and to the designing of suitable interventions. For example, severe and persisting insecurity may perpetuate PTSD symptoms, multiple traumatic losses, separations and material deprivations resulting in prolonged grief, separation anxiety and depression. A perception of injustices on the other hand tends to generate persisting anger.

The Development-based Trauma Framework (DBTF) (26) pointed out that the linear dose-dependent model for understanding posttraumatic impacts is insufficient in explaining the risk for developing psychopathology, and stressed the relevance of non-linear dynamics in a threshold model. Also, the model includes the notion of Cumulative Trauma Disorder (CTD) as a framework for chronic and cumulative effects of trauma, and an alternative for the PTSD concept. CTD was described as a trans-diagnostic cluster which may encompass a wide range of psychopathological phenomena comorbid with PTSD, such as psychosis, dissociation, depressive, anxiety, and somatisation disorders, and memory and executive function deficits.

Building on the previous theories, we suggest the following model for understanding the complexity of posttraumatic responses over the survivor’s life trajectory.

To begin with, we conceptualize mental health of an individual as a balancing act of protective resources and risk factors. Both protective resources and risk factors are rooted in all levels of the ecological and social environment: the micro level (disposition, personality), the meso level (family interaction and support, community, working environment, social life), the exo level (broader social and political environment), and the macro level (spirituality, (sub)culture, belief system, ideology) (27).

As long as the “vulnerability scale” remains balanced, individuals will have a good mental health although they have been impacted by adversities in their lives and may have temporarily presented with symptoms of psychopathology. In case of imbalance of the “vulnerability scale”, the balance can be re-established by receiving interventions focussing on “healing” of the damage (such as psychopathology) and enhancing protective resources, as both types of interventions will strengthen resilience.

When a PTE strikes the ecological environment, it can cause damage on all of its levels. In the following paragraph, we will focus on the micro level only, and comment on the long-term mental health consequences of exposure to PTEs.

Because each individual adjustment following a PTE is the outcome of a unique, cumulative mix of person-centred and socio contextual risks and protective factors (28), four different types of reactions can be observed in survivors (24). The resilient type will experience no or just few symptoms of sub threshold psychopathology. The recovering type will develop initial symptoms and may cross the threshold for the acute stress disorder or the PTSD diagnoses, but this will soon be followed by complete recovery. The delayed type will develop symptoms of distress and even cross the threshold for PTSD when exposed to a PTE, but will not fully recover. Over the life trajectory, the sub threshold PTSD symptoms will at times be present, they show a tendency to worsen over time (7), and evolve into PTSD at a later stage. Research has shown that delayed PTSD seems to endorse prodromal symptoms with a progressive addition of symptoms over time, and intervening stressful events during life precipitate its onset (29). Also, studies have demonstrated that individuals with sub threshold PTSD experience impairment in their daily lives (30). Finally, the chronic type will develop PTSD when impacted by a PTE, and will keep suffering from an ongoing level of distress over time.

The “hidden” impacts of exposure to PTE’s are observed, particularly, in the delayed type of reaction. These individuals may present themselves with different and changing types of symptoms during their life trajectories, and may at times be diagnosed with “full-blown” psychopathology other than PTSD. We hypothesize that the efforts these individuals invest in coping with their subthreshold PTSD complaints may result in the advancement of dissociative states, attachment problems, personality changes, guilt feelings, shame and rage, identity issues, moral injury, damaged core beliefs, and bodily sensations caused by chronic stress activation. However, these psychopathological phenomena are not persistent, like in the complex PTSD (31), but interchangeable, and they may ebb and flow over the course of life. They resemble the trajectories described earlier as the developmental trauma disorder (32) in children with complex trauma histories. These children are given a range of “comorbid” diagnoses, as if they occur independently from the PTSD symptoms, although they mirror the pervasive effects of trauma on child development. The “hidden” impacts are often hidden for both the survivor and the therapist. The survivor is not aware of a causal relation between these impacts and the exposure to PTEs over his/her lifetime.

We hypothesize that both, the resilient and the recovering types, have an adaptive coping and low vulnerability. These characteristics are, among others, rooted in a stable childhood. A recent study (33) supports this notion showing that a child growing up in a supportive family can have lifelong protective effects, whereas a conflicting and unstable one can construct lifelong patterns of pessimistic appraisals and result in increased vulnerability to PTSD symptoms in later life. The same research supported the conception that the same PTE can have positive and negative outcomes, depending on contextual factors. In both types mentioned, resources, as defined in the COR theory, remain available over time. Moreover, resilient individuals have the capacity for generative experiences and positive emotions despite adversity (34). The delayed type is characterized by medium vulnerability and a maladaptive coping, while the chronic type has a maladaptive coping, and a high vulnerability. In these last two types, resources remain scarce over life. Also, the chronic type may present itself with the chronic form of PTSD or with the complex PTSD (31), as in survivors of both early childhood trauma and other traumatic experiences, such as exposure to war and persecution, later in life (35).

Taking the developmental perspective into account, we suggest that individuals with the resilient and the recovering trajectories will maintain their types of adjustment during their life as long as they will not be confronted with an overload of ongoing and chronic stress and they will be able to keep their protective resources. In failing to do so they can develop other psychopathology, and they run the risk of developing the delayed or chronic types of reactions. Earlier research (36) has suggested that high levels of resilience are only present up to a certain point of threat or loss, and when the threshold is reached resilience to stress weakens and even disappears. Accordingly, a linear increase in the risk for PTSD development in relation to increasing frequency of PTE exposure has been documented (37). However, other research (38) suggests that there is more stability than change in resilience over a longer period of time, meaning that resilient individuals may preserve their capacities for resilience even when repeatedly exposed to PTEs during their lives. As the resources required for resiliency are acquired and aggregated across the lifespan, the amount of resources an individual assembles during his/her life is proportionately dependent on their availability (39).

The ongoing stress in an instable environment of the delayed and the chronic types, together with their vulnerability grade, coping styles, and limited availability of protective resources, seems responsible for the maintenance of symptoms over the life trajectory, combined with the decline of resilience by increased intensity or continued trauma exposure (40). Regarding the impact of the ageing process on vulnerability, some studies (41) predict that old age is related to increased vulnerability to PTEs, because of the lack of psychosocial resources together with obstacles to using them (42). Other studies (43) suggest that older survivors may become more resilient and express a posttraumatic growth (44). This growth might be related, over time, to the strengthening of a survivor’s identity through development of a deeper sense of meaning, spirituality and closeness with others.

**Figure 1:**
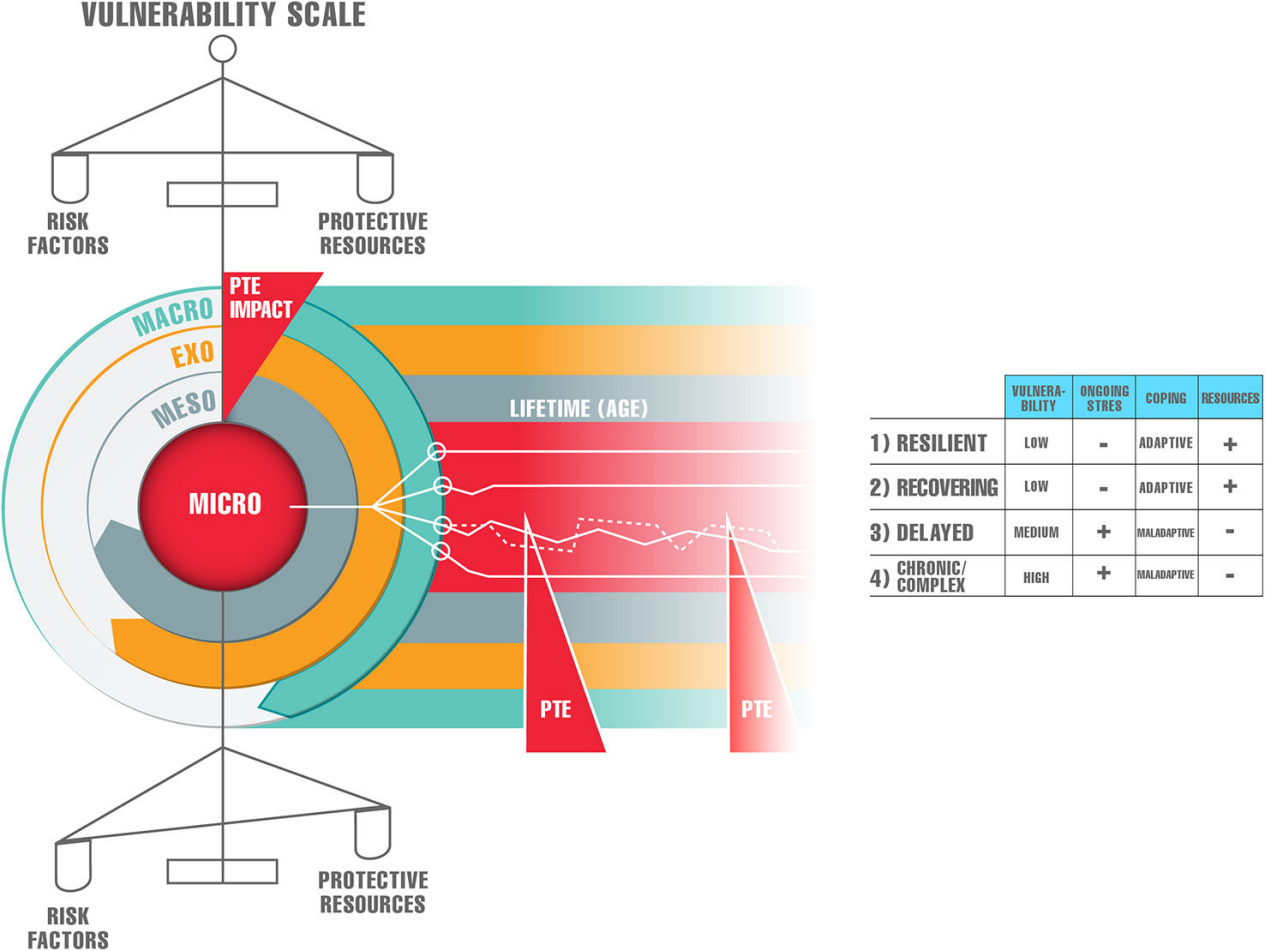
Heterogeneity of long-term impacts of potentially traumatic events (PTEs).

Last but not least, we suggest that the delayed and the chronic trajectories may, ideally, alter towards the recovering one when survivors receive adequate mental health assistance that focuses on the original traumatic experience which tangled the trajectories in combination with interventions directed at strengthening their resilience capacities. This can even be the case when survivors present themselves with other, the “hidden” psychopathological phenomena. We call this the upwards shifting according to the model presented in **Figure 1**. However, this is a time consuming process as a rich reservoir of resources should be built in order to protect the survivor. Since research suggests that the accumulation of resource losses is more rapid and powerful than the accumulation of resource gains over time (39), this endeavour in treatment may sometimes resemble “running against the wind”.

In the following paragraph, we will focus on the delayed type of reaction to PTEs and describe different “hidden impacts” of PTEs on survivors’ mental health.

## Case 1—Rosa: the Lady Who Destroys Biscuits

Rosa was referred by her general practitioner to a psychologist for burnout complaints and psychosomatic symptoms. She is a fifty-five year old woman originating from Azerbaijan and living in the Netherlands since 1994. Her complaints started three years before the referral, when she dropped out of work as a quality officer in a pastry factory. At her workplace, Rosa was very dedicated and often worked long hours. The factory, originally a family business, was taken over by an investor. This led to changes in the corporate culture resulting in exploitation, misbehaviour and loss of quality of the pastry. Rosa was unable to cope with these changes and this led to a halt of her work. She developed stress reactions that manifested themselves in allergies, bad sleep, fluid retention, and weight gain. In addition she also suffered from anger outbursts at unexpected moments, which she found very shameful.

In the initial phase of her treatment, Rosa insisted on speaking about the losses she experienced in the pastry factory out of the conviction that this was the main cause of her complaints. She casually disclosed that she had destroyed biscuits made in the factory in various stores. Although she felt ashamed, she also explained this behaviour as an act of justice, as she found that the quality of the biscuits had deteriorated after she left her work. In this way Rosa expressed her anger and coped with the loss of her workplace. At the time that Rosa dropped out of her job, huge numbers of refugees fleeing the civil war in Syria were entering the Netherlands. They were housed in temporary accommodations and also in her place of residence. Meeting them in the shopping centre and seeing empty looks in the eyes of some of them filled Rosa with horror and physical disgust. Although being a refugee herself, she confessed, she did not have empathy for the newcomers.

In the next sessions, Rosa’s background was carefully explored. She was born in Baku, the capital city of Azerbaijan, in an educated and open minded ethnic Azeri family. She has a two years younger brother. She shared having a normal psychosocial development in her youth and adolescence, and being brought up by caring and loving parents. Her brother moved to Canada in 1987 to study, married a Canadian, got two daughters and build a career in computer business. In the late 1970s, Rosa married Vartan, a now fifty-six year old ethnic Armenian man, also originating from Baku. Vartan too was brought up in a family with a tradition of being open minded. They set out to build a family in a country where ethnically mixed marriages were not commonly accepted. Their only daughter Sofia was born in 1989. Rosa acknowledged that she has a similar character to her, being highly sensitive and open to other people.

In the first sessions Rosa remained aloof while sharing about her past, only starting to report in more detail about her life when she realized that the therapist was well aware of what happened in Azerbaijan in the 1990s. Then she shared that she was working at the police department of Baku as a Special Victims Unit (SVU) detective for five years in the 1980’s, until she was advised by her superior that it would be better for her to quit this expertise. He was concerned and feared that she would drop out with physical and psychological complaints as a consequence of the nature of the work as a SVU detective. At the time she suffered from somatic problems (specifically, allergic manifestations) probably as a result of her detective work in which she was confronted with many horrible scenes of death and violence. Rosa was transferred to the police financial investigation department. Further exploration of the details of her physical condition at that time showed that the physical complaints after dropping out as a SVU detective resembled the current complaints of allergy and other physical ailments. This physical response was for the therapist the first indication that there might be hidden trauma-related problems in Rosa’s past.

Rosa, together with the therapist, continued exploring her past in greater depth. In January 1990, at the time of the breakup of the Soviet Union, pogroms against Armenians broke out in Baku. Being a police officer, Rosa had officially the task to intervene in cases of breaking the law, but at the same time she was ordered by her superiors to stay at a distance while witnessing Armenian compatriots being molested, tortured and even killed by organized gangs. Because of these pogroms, her husband and his family were forced to leave to the neighbouring country of Georgia, leaving Rosa alone with her then one year old daughter. Due to communication problems, as there were no cell phones and internet back then, Rosa and her husband lost track of each other. Rosa, unsure of her husband’s whereabouts, also decided to leave Azerbaijan in the course of 1990, and joined her brother and his family in Canada. She was dedicated to build a new live with her daughter Sofia. The migration brought a lot of uncertainty with, she coped with it by working as many hours as possible, and eventually succeeded in building a successful career as a manager at a fraud prevention institute. After eleven months of residing in Canada, she learned *via* the Red Cross tracing agency that Vartan was alive and was, in the meantime, accepted as a refugee in the Netherlands. In the following 4 years, they visited each other and travelled back and forth between the Netherlands and Canada. Eventually they decided, after much deliberation and thinking, to reunite as a family in the Netherlands. Rosa left her newly attained world in Canada, her good job and career prospects, for an uncertain life of a migrant in the Netherlands. Initially, she set out as mother, raising their daughter, and learning the Dutch language from other mothers “at the fence of her daughter’s schoolyard”. This lifestyle prompted complaints of gloom and brought back unwanted memories of Rosa’s past in Baku. However, when the family moved to their current place of residence, she found a job at the pastry factory, where she soon evolved to the status of quality officer and functioned well prior to the takeover by the investor. She could work hard again and be less preoccupied with her past. Simultaneously with dropping out of her job, Rosa was also confronted with the empty looks in the eyes of Syrian refugees. These reminded her of the looks she encountered in the victims of the pogroms in Baku. In the therapy sessions Rosa started to explain in more detail about the events she witnessed in that period, including finding the mutilated corpse of her former violin teacher murdered by the looting gangs.

Well into the therapy, Rosa opened up about her worries, and revealed her shame and guilt about her ethnicity in relation to what had happened in Azerbaijan. She therefore remained very reluctant towards the Armenian community in the Netherlands despite her mixed marriage. It also became clear that Rosa and Vartan never spoke about the consequences of the political turmoil in their home country on their lives. This was a silent agreement between them.

In terms of this article, Rosa is a good example of the “hidden” impacts of trauma. She was referred with unexplained physical complaints and a burnout, implying that a standardized cognitive behavioural therapy aiming at reduction of these complaints should be applied. Only by careful exploration of her life trajectory, building trust by showing familiarity with her background, and taking time to explore her past in detail, a link between her trauma-related background and her current complaints could be established. Several PTEs had a great impact on Rosa’s life. Her work as a SVU detective appeared to be the core of her current somatic complaints (allergic reactions), and an individualized expression of the “the body keeps the score” concept. Also, her experiences with the pogroms in Baku, the memories she thought she had left behind, were kept covered for many years by working as many hours as possible. Her work as a police officer during pogroms, as this was laden with guilt and shame, was connected to her current isolation from her fellow citizens in the Netherlands. Relocation from Canada to the Netherlands and the loss of her professional career for the second time were also experienced as stressful life events causing imbalance on the vulnerability scale. Finally, she never spoke with Vartan about the turmoil in Azerbaijan as a manner to deal with its impacts.

In the treatment, Rosa steadily developed insights into the intertwining of her complaints and her background, and was involved in the process of giving meaning to her current suffering. Because of the strong relation between the PTEs and her physical complaints, Somatic Experiencing (SE) therapy was applied later on, with the aim to learn to tolerate and understand her physical experiences. SE was combined with individual client centred psychotherapy and EMDR. The treatment resulted in a significant reduction of Rosa’s physical complaints and her sleeping problems. Her anger at the refugees in her residential town also diminished, and Rosa stopped destroying biscuits in the stores.

## Case 2—Peter: the Former Ngo Worker on a “Warpath”

Peter is a sixty-four year old man who was referred for treatment of mood complaints. He was a field professional at different non-governmental organisations (NGO’s), and led many missions, such as to war-torn Bosnia and Cambodia, and in the aftermath of the Tsunami in Thailand in 2004. In his professional career he experienced several life-threatening situations and was confronted with death in different forms. He lost colleagues due to accidents, murder and suicide. However, at the start of the therapy Peter does not report being burdened by these experiences and losses. First and foremost, he describes his anguish over bureaucracy and courses of action that he did not agree with, and that were taken at the time he was in the field. He preferred working intuitively, and from the view of trying to understand a crisis situation from a local perspective, a method that proved to be conducive. He felt a strong connection with his team members, but ran into conflict with his superiors about everlasting bureaucracy and, in his opinion, wasting of funds. As the years went by, bureaucracy got worse instead of better and Peter found it increasingly difficult to find his place in the organization. He stopped working three years before the start of the treatment. Since then he follows the news about NGO’s fervently and still gets angry about how crises all over the world are tackled by them. At the same time, a mixture of positive and negative memories related to his working past are triggered. This confuses him and makes him sad. He also misses the meaningfulness of his professional life.

Peter suffers from mood swings and anhedonia already for half a year, he is often at home, and has trouble absorbing the daily routine. However, he still enjoys spending time with his grandchildren. His appetite is normal, and he does not report having sleeping problems. In his whole life he has never experienced nightmares or flashbacks related to the missions and the threatening life events he has faced. Sometimes he suffers from irritability and can have an outburst of anger, mostly when he perceives that injustice is done.

In the first sessions Peter was diagnosed with a persistent depressive disorder arising from excessive thinking and worrying about the predicaments linked to his professional career. He was, in particular, wrestling with many questions and moral dilemmas connected to his work at the NGO’s. These can be framed within the moral injury concept, defined as “the enduring psychological, biological, spiritual, behavioural and social consequences of perpetrating, failing to prevent, bearing witness to, or learning about acts that transgress deeply held moral beliefs and expectations” (45, p. 697).

In the following sessions, more attention was paid to Peter’s early psychosocial development in which no irregularities were found. Peter inherited a strong sense of justice from his upbringing. This brought him already at a young age into conflict with teachers and other authorities. However, this oppositional behaviour was not pathological to the extent that it would have met criteria for a personality disorder. Rather, it seems to be a character trait which causes that Peter is not always liked by others because of his criticism, but it does not appear to play a negative role in his family life and in relation to close friends. While recalling certain events linked to the missions, it was noticed that Peter would sometimes show strong emotions, mostly sadness and tears. However, these emotions would dissolve within seconds, morphing into expressing his anger about certain bureaucracy issues surrounding these events. This formed an indicator that “hidden” problems were present and they did surface later on in the treatment. Peter inclines to ignore and suppress his emotions in contact with people, mostly by moving a discussion to another topic, a phenomenon that often occurred in the sessions. Afterwards when he is alone, he rethinks the topics of a conversation, and only then he starts experiencing the corresponding feelings often resulting in rumination and a depressed mood.

Further in the treatment, his thoughts and ideas about certain events during the missions were explored more in-depth. Through this process, it became apparent that Peter suffered both from moral issues regarding politically motivated decisions and acting of his superiors which were not always in the interest of the local population, and from the shocking events which he had personally experienced. In this probing process Peter also began to realize that his way of coping showed similarities with the coping mechanisms of the people he reached out to as the NGO professional, survivors that showed no emotions in the middle of a catastrophe and just went about their daily business. Peter came to realize that his work confronted him with seriously impacting life events. He dealt with the impact of these events by comparing himself to the victims and labelling his own experiences as insignificant in comparison with theirs. When Peter came to full realisation of this defence mechanism, he unexpectedly developed sleeping problems, nightmares and re-experiencing of the work related events (PTEs). At that point in the treatment, Peter underwent for the first time emotions related to the events experienced in the past. It was an enlightening and cathartic experience for him. Nevertheless, it also made him upset because he realised that he was about to develop a full blown PTSD, a condition which he thought he will never suffer from.

Peter experienced numerous PTEs. The loss of several colleagues seemed to have had the strongest impact on him as many of them became close friends over the years. The knowledge that the suicides of his colleagues were a result of their inability to cope with constant horrors, was something Peter didn’t want to face or think about until he was confronted with his avoidance in the therapy. He came to acknowledge that this fear came too close to his own anxiety related to losing control and committing suicide. Realizing that his own emotions were genuine and authentic responses to extreme experiences he had faced, helped Peter to find balance again. The depressive feelings were reduced to a great extent. Further, psycho education regarding the moral injury concept helped him realize that his anger, originating from the moral injuries, protected him from strong emotions linked to many personal losses over the last decades. Engaging into fights and disputes with his superiors was, even though justified in terms of content, an emotionally inadequate way of coping with the horrifying experiences and losses. The PTSD symptoms which emerged in a course of the therapy were successfully treated with EMDR.

Peter is an example of the delayed type of trajectory, where a lack of giving meaning to experiences, in combination with maladaptive emotional coping and withdrawal from active work, led to evolving depressive symptomatology and, temporarily, to a full-blown PTSD. Moral issues played a significant role in the emergence of Peter’s mood complaints, as he was coping with consequences of political decisions impacting his daily work, and with feelings of powerlessness to influence them. However, frustration about the moral dilemmas also acted as a camouflage for authentic and painful emotions arising from multiple life threatening experiences he had accumulated over time.

Peter did not develop a PTSD earlier in life, even though he was confronted with many PTE’s. PTSD symptoms developed only when Peter became aware of his emotional coping style. It remains an open question whether Peter would have developed PTSD at a later stage in his life without being triggered by the treatment? However, it remains the fact that Peter got referred because of suffering from mood complaints and a poor quality of life, something that changed significantly for the better at the end of the therapy.

## Case 3—Behrooz: the Aggressive Iranian Man With Attention Deficit Hyperactivity Disorder (Adhd)

Behrooz is a twenty-seven year old man originating from Iran and residing as a refugee in the Netherlands. He was referred by a general practitioner with complaints of impulsivity and aggression outbursts. He was suffering from these problems for many years, but has not sought help earlier. The practitioner suspected Behrooz from ADHD, and asked for diagnostics and treatment. At time of the referral, it was unknown how and when Behrooz ended up in the Netherlands. The only information available was that he had lived illegally in the country for a long time, and that a residence permit was granted to him four years earlier, when the immigration department legalized all illegal immigrants through a general pardon.

During his first interviews, Behrooz stood out because of his loud speaking and agitated behaviour. He was, in particular, angry about the way the Dutch society and politics had treated him. As Behrooz gained more confidence in the therapist, and realised that he was not unfamiliar with the lengthy and impersonal asylum procedures and their impacts on an individual, he started to disclose more about his remarkable past.

Behrooz was born a few years after the Islamic revolution in Iran. His family was liberal and had been politically active for generations. As the youngest son, Behrooz started to participate in their political activities already at a very young age. He attended secret meetings and helped distributing leaflets in the streets. At the age of twelve, he was arrested and jailed by the Iranian secret service. He was brutalized and tortured for days, until he was released because his family had paid a ransom. Shortly after his release, the family arranged for Behrooz to leave Iran illegally and escape to neighbouring Turkey *via* the mountains. This turned out to be a frightening experience because he got lost on the way, and failed to meet the contact person who would guide him across the border.

In the therapy sessions, Behrooz denied being bothered by these events. He stated repeatedly that so many people from Iran had similar experiences. He felt he was no exception and therefore should not be bothered by them. In Turkey, he eventually found domicile with an older man who gave him odd jobs, such as collecting scrap metal and selling it, in exchange for food and lodging. In company of this man Behrooz found protection and a sort of mentorship. This was important as he was still young, separated from his family, and illegally in the country with no rights whatsoever. Behrooz quickly got streetwise, learned about how to survive, and became a specialist in small businesses and arranging odd jobs for others.

At the age of sixteen, his family in Iran informed him that the Netherlands has a separate asylum procedure with extra protective rules for minor refugees and that within this procedure his chances to be recognized as a refugee were substantial. They advised him to leave Turkey and join an aunt who was already living in the Netherlands for more than ten years. On arrival and asking for asylum, Behrooz was placed in a small-scale shelter, got a mentor and went to school, where he learned Dutch fluently in a short period of time. However, his asylum procedure did not fare well. The immigration authorities did not believe his narrative of flight and his request for protection, partly because of the young age at which he claimed to have been politically active and because of the length of time he had spend in Turkey.

As a consequence, Behrooz was forced to leave the shelter immediately after his eighteenth birthday and, yet again, forced to find his way in illegality. He lived as a squatter for five consecutive years. He was seriously disappointed and outraged, as this situation reminded him of the time he had spend in Turkey. Moreover, he had developed different expectations about his future in the period he was accompanied as a minor asylum seeker. At the age of twenty-three Behrooz was finally granted a residence permit within a general pardon. He expected to be happy, but on the contrary felt seriously disappointed in himself and what he had achieved in life. Due to illegality he could not complete a vocational training and was obliged to find a layman’s job as quick as possible or all his social benefits would be stopped.

When the therapist inquired about his complaints, Behrooz indicated that he was mainly concerned about the fact that he had so little self-control and that he could rapidly become extremely verbally aggressive to other people. He felt very guilty and ashamed about it. In these outbursts he would accuse the other of being unreliable, lying and intentionally aiming to harm him. He added that being out of control also terrified him, because he had not any recollection of what had been said by himself or by others afterwards. This led to the hypothesis that he experienced dissociative phenomena (which was later confirmed in the therapy). The suspected ADHD was confirmed by a diagnostic interview, and extensive inquiries were made on PTSD. However, Behrooz denied suffering from nightmares or other re-experiencing phenomena. He insisted on being angry over the particular period in his life when he fought for the residence permit and felt abandoned by the Dutch society and the minister of immigration.

The first intervention in a course of the treatment was to prescribe Behrooz a psycho stimulant (Dexamfetamine). This lead to reduction of the sensation of chaos in his head and a better control of emotions. Medication also caused that he became more accessible for constructive exploration of his life and emotions as he no longer got overwhelmed by anger during the sessions.

Eventually Behrooz started to share his experiences from Iran and Turkey, and admitted for the first time that he has also occasionally suffered from nightmares for many years. As it turned out, he consciously wanted to keep that information to himself, because he developed a personal theory about the function of his nightmares. He suffered from them from the age of twelve, and believed that the nightmares were a way of dealing with his past. He also had the expectation that they would disappear when he worked through them. After re-evaluation of his complaints, the therapist concluded that Behrooz fulfilled criteria for the PTSD diagnosis and an inventory was made of his traumatic experiences.

Behrooz received psycho-education about the symptoms of PTSD, such as hyper arousal and its similarities and differences with hyperactivity and impulsivity complaints of ADHD. These symptoms are often difficult to distinguish in patients with both disorders, especially in those who have been traumatized at a young age. In the follow-up sessions, the traumatic recollections were treated with EMDR. As a result, the nightmares disappeared and Behrooz also reported changes in his state of hyper alertness, a phenomenon he was never aware of as he had grown up with it and did not know better. After the trauma-focus treatment, Behrooz continued with his therapy and contributed a personal signification to his experiences. He redefined the survival skills he mastered in the forming periods of his life to personal capacities. Behrooz achieved a so-called posttraumatic growth. He began to create a newly balanced personal and professional future in which he started a vocational training as an electrician. The therapist played hereby an important role, advocating for Behrooz with the local government which paid the costs of the training.

The rationale for development of specific symptoms in the course of Behrooz’s life only became apparent when the therapist was able to build a trust relationship and together with Behrooz carefully and patiently map his personal history. He originated from an action-oriented family, had an inherited disposition to ADHD, and was tortured and traumatized at a very young age, leading to the evolution of survival strategies and dissociative phenomena of which he himself was unaware. As it showed during the therapy, his way of living was dictated by coping with PTSD with dissociative characteristics, expressing themselves in impulsivity and aggression. From the moment he got a residence permit, Behrooz relaxed and had more time to think and reflect on his life. Disappointment about what he had achieved dominated his thoughts, and the unanticipated continuation of his impulsivity and aggression drove him to find help. Although Behrooz has already made great progress in his therapy, the process is still not completed as the psychological damage inflicted at a young age demands prolonged consolidation and help in shaping a stable personal and professional future for himself.

## Treatment Implications

The life trajectory of survivors with the delayed type of reaction to aversive events resembles a journey through a “field of landmines”, as these individuals seem to have to walk on their toes throughout life in an attempt to cope with psychological and other impacts of exposure to ongoing PTEs. These survivors try to avoid feeling the essence of being profoundly changed by the impacts of their traumatic experiences: feeling powerless and abandoned, losing control over their existence, and in the most extreme cases experiencing the “soul murder” (46), the breakdown of the most basic foundations of humanness. As exposure to trauma may have changed their core beliefs, and survivors start to experience the world as malevolent, meaningless, and anxiety provoking, they develop ways of avoiding this anxiety, many of which are likely problematic on a long term (47). Since these problematic behavioural and cognitive coping strategies fail to protect survivors from anxiety on the long term and keep them from integrating the trauma in their autobiographical memory, survivors’ negative self-appraisals relating to the original trauma event and/or subsequent coping attempts become more prominent, creating a vicious circle and provoking more anxiety and feelings of unsafety. After years of struggling to maintain psychological balance at a high cost, these individuals may finally decompensate and develop the “hidden” psychopathological impacts of trauma. Eventually, given the accumulation of PTEs over life together with failing of adaptive protection mechanisms, these survivors may further deteriorate and develop PTSD with delayed expression later in life. In other words, these individuals wrestle with the impacts of traumatic experiences for a long time. They seem to be relatively unharmed by them to the point of no return. Then, they crush under the allostatic load (48) leading to development of physical and emotional damage, and pay a toll for functioning in a “survival mode” for years.

We suggest that the core aims of all psychotherapeutic interventions for survivors of war and violence are to help them to regain control over their lives, restore self-efficacy and a sense of agency, reattach with humanity, give meaning to traumatic experiences and suffering, and regain hope for the future. These therapy aims go beyond the goals of simply reducing symptoms of PTSD, depression, and other comorbid conditions, although reduction of symptoms and associated suffering are important. More material on the fundamental ingredients of establishing and maintaining a fruitful therapeutic relationship with survivors of war and violence can be found elsewhere (49).

In cases where clients present themselves with the “hidden impacts”, and where a delayed trajectory has being established through a careful anamnestic examination of life stressors, traumatic events, sources of resilience, and coping efforts over their lifespan, the “original” traumatic experience should first be identified. The next step should be to establish causal relationships between the index trauma and other psychopathology a survivor has suffered from later in life. Through this process, a shared explanatory model is created by the therapist jointly with the survivor, as this will help to give meaning and significance to a survivors’ lifelong struggle with the trauma impacts from a developmental perspective. The current mental health problems are conceptualized as a reflection of the survivors’ psychological imbalance, a mirror of their lifelong dynamic struggle between sources of resilience and damage. In the assessment phase, clinicians should be guided by the string of causation principle in understanding the development of mental health problems. This obliges them to pose the question why a survivor has developed a certain type of psychopathology at a specific moment in life, what protected the survivor from suffering earlier in life and which, previously present and effective, protective sources can be strengthened in order to help the survivor to restore balance of the “vulnerability scale” (49)?

Generally, the next move in treatment should be to identify and prioritise interventions which have to be applied. Mostly these are actions towards minimizing the impact of current stress, and/or those aiming at strengthening protective resources. Where necessary, psychotropic medication can be introduced in order to enhance control over current symptoms. The aim of these interventions is to direct the “vulnerability scale” closer to a balance.

Further, trauma-focus treatment targeting the index traumatic experience can be applied. All of the evidence-based trauma-focus approaches currently recommended (Narrative Exposure Therapy (NET), Eye Movement and Desensitization and Reprocessing (EMDR), Cognitive Behavior Therapy (CBT)) (49, 50) include exposure and cognitive restructuring components, while Somatic Experiencing (SE) (51) and Sensorimotor Psychotherapy (52) target traumatic memories rooted in the body. All these approaches can be intertwined with cognitive interventions aiming at providing a meaning to the impacts of traumatic experiences, and helping survivors to deal with issues of shame, guilt and moral injury. The process of giving meaning to one’s life trajectory should create congruency between survivor’s global (core beliefs) and situational (initial appraisal of a traumatic event) meanings in terms of beliefs and goals. This is important in order to stop persistent rumination about the impact of a traumatic experience (53). Ideally, positive outcomes of this process are developing of new connotations, new coping skills, including cognitive coping skills, problem-solving and help-seeking skills, ability to control and regulate affect (54), and installation of a sense of hope for the future, dignity and coherence (55). Last but not least, as context enveloping the treatment process may change over time, it is important for the therapist to acknowledge these environmental impacts on the treatment strategies applied, and to keep shifting back and forth between the interventions aiming at reducing posttraumatic damage and those strengthening resilience capacities.

## Limitations and Future Research Agenda Suggestions

This article presents a theoretical model for understanding the long-term impacts of exposure to PTEs. It also discusses the mental health consequences of psychological trauma beyond the PTSD diagnosis, and suggests an alternative treatment approach to the “hidden” impacts. This article is based on clinical experience with several hundreds of survivors from Europe, Middle and Far East, Africa and the Caucasus region treated in outpatient settings in the Netherlands over the past two decades. Therefore, the main limitation of this article is a lack of sound scientific research supporting the suggested concepts and ideas.

However, data supporting different aspects of the theoretical models presented and discussed in this article are already available, but it seems virtually impossible to test all components of comprehensive models all at once. Studies that have examined the latent structure of PTSD symptoms using taxometric analyses have supported a dimensional structure (56–58). Other research (59, 60) has identified multiple, unique trajectories of adjustment in the aftermath of exposure to a PTE on the short and long term (61), showing that the availability of resources and the change in resources resulting from highly aversive life events play a crucial role in human adaptability to extreme stress.

Future research should in depth focus on the longitudinal trajectories of those impacted by PTEs taking into account the complex ecological and environmental factors that shape these trajectories. Therefore, trials should also include a local, emic understanding of trauma and psychological distress. Moreover, symptom outcomes should be expanded beyond PTSD and depression to include a range of comorbid and/or multidimensional “hidden” symptom patterns that can be observed in clinical work with the survivors. The non-symptomatic impacts, like changes in quality of life, resilience, social integration, behaviour (such as aggression) and inter personal relationships deserve more focus in future studies. The suggested treatment approach to the “hidden” impacts of PTEs should be tested by a combination of empirical and clinical knowledge with the ambition to ultimately find which blend of interventions might be the most effective, taking into account sampling and contextual factors that can influence outcomes.

## Summary

Impacts of exposure to war and violence are heterogeneous, and several different lifetime trajectories can be observed in the survivors. Moreover, the survivors can, on the long term, present themselves with PTSD and/or with a wide array of other psychopathological phenomena. These phenomena are not persistent, but they fluctuate over time, and are not always easy to link to the index trauma according to the string of causation principle. However, establishing a causal relation between the index trauma and the “hidden’’ impacts seems to be the first step in assisting survivors with their complaints. Further, we suggest that treatment of the index trauma leads to remission of the ‘‘hidden impacts”, and together with strengthening of resilience sources and minimizing of the current life stress, to reestablishment of the survivors’ mental health balance.

## Author Contributions

BD, JR, and AM-J have all equally participated in writing the article. It should be noted that the case presentations are based on real clients, but modified for the purpose of respecting their privacy.

## Conflict of Interest

The authors declare that the research was conducted in the absence of any commercial or financial relationships that could be construed as a potential conflict of interest.

The handling Editor declared a past co-authorship with one of the authors BD.
